# Supplementary material for: Developmental plasticity of cardiac anoxia-tolerance in juvenile common snapping turtles (Chelydra serpentina)
Source: Proc Biol Sci. 2019 Jun 26;286(1905):20191072. doi: 10.1098/rspb.2019.1072 (PMC6599983; doi:10.1098/rspb.2019.1072)
Supplement: Supplemental Materials & Methods [file rspb20191072supp1.pdf]

1. Supplementary Materials and Methods

(a) Turtle collection, incubation, and husbandry

Snapping turtle (*Chelydra serpentina*) eggs were collected from the wild in Minnesota, USA, and transported to the University of North Texas for incubation. Permission to collect the eggs was granted to DA Crossley by the Minnesota Department of Natural Resources (permit no. 21232). Two eggs from individual clutches were staged to determine age. Incubations lasted no more than 55 days and all eggs were maintained at 30°C, a female-determining temperature [1-3]. Eggs were embedded to their midpoint in vermiculite, inside plastic incubators (2.5-L Ziploc Container, SC Johnson, Racine, WI, USA) that were stored in a walk-in Percival Environmental Control Room (model IR-912L5; Percival Scientific, Perry, IA, USA). The vermiculate was mixed in a 1:1 ratio with water, as previously described [4].

At approximately 20% development (9-12 days after laying; determined by embryonic staging), eggs were randomly assigned to either the normoxic/atmospheric oxygen (21% O_2; designated as N21) or hypoxic (10% O_2; designated as H10) cohorts. To achieve the desired oxygen level, parallel gas inflow and outflow tubes were attached to the incubators and O_2 gas mixtures were set at a flowrate of 2–3 l min^{-1} using rotameters (Sho-Rate Brooks Instruments Division, Hatfield, PA, USA) downstream of either compressed N_2/air mixture or air alone. The gas mixtures passed through an H_2O bubbler to ensure 80–95% relative humidity and their compositions were monitored continuously with an oxygen analyser (S-3AI; AEI Technologies, Pittsburgh, PA, USA).

Upon hatching, all turtles were housed in common, normoxic (21% O_2) conditions at 26°C. Turtles were fed dry crocodilian food (Mazuri, PMI Nutrition International, Brentwood, MO, USA) 2-4 times weekly, *ad libitum*, and kept in a daily 12:12 light-dark cycle. After 7 months, the hatchlings were transported by air-freight to the University of Manchester, UK. Hatchlings were individually housed in normoxic conditions at room temperature and fed fresh krill and Tetra ReptoMin (Spectrum Brands, Blacksburg, VA, USA), *ad libitum*, 3x weekly. Turtle husbandry and experimental procedures were carried out in accordance with University of Manchester handling protocols which adhere to the United Kingdom Home Office legislation.

(c) Cardiomyocyte isolation

Experimental procedures were conducted on juvenile turtles aged between 15 and 24 months (357.31 ± 50.01 g). Ventricular cardiomyocytes were isolated by enzymatic dissociation, as previously described [5]. Briefly, turtles were euthanized by rapid decapitation, followed by brain destruction. The heart was excised and cannulated through the aortic arch into the ventricle. The ventricle was initially perfused with isolation solution for 8-10 min (to remove blood and debris) and then with enzymatic dissociation solution for 26-30 min (see Table 1 for solution recipes). Solutions were heated to 28-30°C with an In-line Heater/Cooler Peltier (model SC-20, Warner Instruments, Hamden, CT, USA), connected to a Single-Channel Temperature Controller and monitor (model TC-324C, Warner Instruments). After perfusion, the ventricle was removed and minced into small pieces (≤3 mm) and individual ventricular cardiomyocytes were released by gentle agitation. Finally, myocytes were suspended in isolation solution and maintained at room temperature for up to 8 hours.

(d) Morphometric analysis

Myocyte morphometrics were determined with confocal microscopy (model LSM 7 Live, Zeiss, Cambridge, UK). Freshly isolated cells were incubated with 1% wheat germ agglutinin (WGA; Alexa Fluor 488 conjugate, ThermoFisher), in PBS, for 60 min at room temperature. Consecutive plane scans (x-y) were made through the cell to make a three-dimensional model (Z stack), from which cell length, width, depth, and volume were calculated using the Zen imaging software (Zeiss).

(f) Experimental protocol

Cardiomyocytes were loaded, with the AM-ester, cell-permeant fluorescent indicators (Invitrogen, Loughborough, UK) Fura-2 AM, BCECF, and dihydroethidium (DHE) to measure $[Ca^{2+}]_i$, pH_i, and ROS, respectively, at room temperature. $[Ca^{2+}]_i$ and pH_i were measured simultaneously, by co-loading cardiomyocytes with Fura-2 (0.075 µmol l^{-1}) for 10 min and BCECF (0.8 µmol l^{-1}) for 30 min. ROS were measured separately, by loading cardiomyocytes with DHE (5 µmol l^{-1}) for 30 min. Following the loading protocol, cells were resuspended in fresh isolation solution for 15-20 min to allow de-esterification. Cardiomyocytes were then placed in the recording chamber (at room temperature), perfused with normoxic saline, and stimulated to contract at a frequency of 0.2 Hz. We chose this stimulation frequency because it lies well-within the normoxic range of common snapping turtles (10-25 BPM [6, 7]), as well as the estimated anoxic range (5-12 BPM, based on estimates from anoxic *Trachemys scripta* [8]). While we acknowledge that our stimulation protocol does not recapitulate the effects of *in-vivo* anoxia, where turtle heart-rate is reduced by 50%, we chose to keep this parameter constant, so we could interpret our findings in the absence of any changes in frequency, which are known to independently affect contractility and calcium [9]. After a 10-min stabilization period, the perfusate was switched to an anoxic saline for 20 mins, and cells were subsequently reoxygenated, by switching back to the normoxic saline for a further 10 mins (Fig. 1A). Oxygen was measured continuously throughout the protocol and was undetectable during the anoxic period.

(g) Fluorescent imaging

All excitation light waves were filtered with a Nikon T510lpxru dichroic (Chroma Technology Corp, Olching, Germany) long-pass filter and emitted light waves were collected using HQ535/50m (Fura-2 and BCECF) and ET585/40m (DHE) emission filters (Chroma). Fura-2 and BCECF were excited sequentially at 340/380 nm and 490/440 nm, respectively, and light was collected at 515 nm. DHE was excited continuously at 490 nm, with the addition of an ET480/40x excitation filter, in combination with the dichroic, and light was collected at 605 nm. Fura-2 and ROS input and exit slit widths were each set to 10 nm and BCECF slit widths were set to 7 nm (input) and 4 nm (exit). Fura-2 and BCECF were calibrated using previously published protocols [10-12]. For the BCECF calibration, cells were perfused with a series of solutions of increasing pH (6.5 → 8.0) that contained nigericin (7 µmol L^{-1}), a K^+ ionophore (Table 1). A four-step pH calibration was used to generate a standard curve, in which the fluorescence output measurements were converted into pH_i. For the Fura-2 calibration, cells were perfused with an extracellular solution (Table 1) that contained the metabolic inhibitors rotenone (2 µmol L^{-1}), Na-iodoacetate (5 µmol L^{-1}), carbonyl cyanide 3-chlorophenylhydrazone (5 µmol L^{-1}) to avoid sequestering Ca^{2+} in the mitochondria, and ionomycin (10 µmol L^{-1}; a Ca^{2+} ionophore). To measure R_{max} and R_{min}, EGTA (10 mmol L^{-1}; a Ca^{2+}-chelating agent) and $CaCl_2$ (2 mmol L^{-1}) were added to the extracellular solution, respectively. The collected 340/380-nm ratiometric data were converted to $[Ca^{2+}]_i$, using the K_d values and the equation described by Lattanzio [11]. Briefly, K_d values were derived from a Ca^{2+}-binding standard curve for Fura-2, using solutions of varying pH (7.4 → 5.5, at 22°C), from the Lattanzio study, and by producing a best-fit curve, which shows a gradually lower Ca^{2+}-binding affinity (i.e. a higher K_d) with decreasing pH. Finally, $[Ca^{2+}]_i$ was calculated with the formula $\left[Ca^{2+}\right]_i = K_d \times \left[\frac{(R-R_{min})}{(R_{max}-R)}\right] \times \left(\frac{S_{f2}}{S_{b2}}\right)$ [11].

Table S1. Composition of salines and solutions.

Component	Isolation solution	Dissociation solution#	Normoxic saline¶	Anoxic saline‡	BCECF calibration	Fura-2 calibration$
NaCl, mmol l^{-1}	100	100	95	82		95
KCl, mmol l^{-1}	10	10	2.9	2.9	140	2.9
$NaHCO_3$, mmol l^{-1}			35	15		
Na_2HPO_4, mmol l^{-1}			0.74	0.74		0.74
NaH_2PO_4, mmol l^{-1}			0.6	0.6		0.6
KH_2PO_4, mmol l^{-1}	1.2	1.2				
$MgCl_2$, mmol l^{-1}			1.2	1.2	1	1.2
$MgSO_4$, mmol l^{-1}	4	4				
$CaCl_2$, mmol l^{-1}			2	2		2$
NaLactate, mmol l^{-1}				30		
HEPES, mmol l^{-1}	10	10			10	10
Taurine, mmol l^{-1}	50	50				
Glucose, mmol l^{-1}	20	20	5	5	8	5
EGTA, mmol l^{-1}					1	10$
Nigericin, µmol l^{-1}					7	
Rotenone, µmol l^{-1}						2
Iodoacetate, mmol l^{-1}						5
CCCP, µmol l^{-1}						5
Ionomycin, µmol l^{-1}						10
Trypsin, mg ml^{-1}		0.5				
Collagenase, mg ml^{-1}		0.75				
BSA, mg ml^{-1}		0.75				
$O_2/CO_2/N_2$, %			21, 3, 76	0, 11, 89		
pH	6.9*	6.9*	7.72¶	6.8‡	6.5, 7, 7.5, and 8*	7.7*

*pH adjusted with KOH. #Enzymes were dissolved in the solution, just prior to enzymatic perfusion. ¶Saline was equilibrated with a gas mixture of 3% CO_2, 21% O_2, and 76% N_2, to yield a pH of 7.72 in solution. ‡Saline was equilibrated with a gas mixture of 11% CO_2 and 89% N_2, to yield a pH of 6.8 in solution. $Two solutions were used to calibrate Fura-2 (one contained EGTA and the other contained $CaCl_2$), as described in detail in the Materials and Methods section.

References

1. **Eme, J., Rhen, T., Tate, K. B., Gruchalla, K., Kohl, Z. F., Slay, C. E. & Crossley, D. A., II**. 2013 Plasticity of cardiovascular function in snapping turtle embryos (*Chelydra serpentina*): chronic hypoxia alters autonomic regulation and gene expression. *Am J Physiol Regul Integr Comp Physiol* **304**, R966-979. (DOI:10.1152/ajpregu.00595.2012).
2. **Yntema, C. L.** 1968 A series of stages in the embryonic development of *Chelydra serpentina*. *J Morphol* **125**, 219-251. (DOI:doi.org/10.1002/jmor.1051250207).
3. **Alvine, T., Rhen, T. & Crossley, D. A., II**. 2013 Temperature-dependent sex determination modulates cardiovascular maturation in embryonic snapping turtles, *Chelydra serpentina*. *J Exp Biol* **216**, 751-758. (DOI:10.1242/jeb.074609).
4. **Crossley, D. A., II & Altimiras, J.** 2005 Cardiovascular development in embryos of the American alligator, *Alligator mississippiensis*: effects of chronic and acute hypoxia. *J Exp Biol* **208**, 31-39. (DOI:10.1242/jeb.01355).
5. **Shiels, H. A., Vornanen, M. & Farrell, A. P.** 2000 Temperature-dependence of L-type Ca^{2+} channel current in atrial myocytes from rainbow trout. *J Exp Biol* **203**, 2771-2780.
6. **Frische, S., Fago, A. & Altimiras, J.** 2000 Respiratory responses to short-term hypoxia in the snapping turtle, *Chelydra serpentina*. *Comp Biochem Phys A* **126**, 223-231.
7. **Wearing, O. H., Conner, J., Nelson, D., Crossley, J. & Crossley, D. A., II**. 2017 Embryonic hypoxia programmes postprandial cardiovascular function in adult common snapping turtles (*Chelydra serpentina*). *J Exp Biol* **220**, 2589-2597. (DOI:10.1242/jeb.160549).
8. **Stecyk, J. A. W., Bock, C., Overgaard, J., Wang, T., Farrell, A. P. & Portner, H. O.** 2009 Correlation of cardiac performance with cellular energetic components in the oxygen-deprived turtle heart. *Am J Physiol Regul Integr Comp Physiol* **297**, R756-768. (DOI:10.1152/ajpregu.00102.2009).
9. **Antoons, G., Mubagwa, K., Nevelsteen, I. & Sipido, K. R.** 2002 Mechanisms underlying the frequency dependence of contraction and $[Ca^{2+}]_i$ transients in mouse ventricular myocytes. *J Physiol* **543**, 889-898. (DOI:10.1113/jphysiol.2002.025619).
10. **James-Kracke, M. R.** 1992 Quick and accurate method to convert BCECF fluorescence to pH_i: calibration in three different types of cell preparations. *J Cell Physiol* **151**, 596-603. (DOI:10.1002/jcp.1041510320).
11. **Lattanzio Jr, F. A.** 1990 The effects of pH and temperature on fluorescent calcium indicators as determined with Chelex-100 and EDTA buffer systems. *Biochem Biophys Res Commun* **171**, 102-108. (DOI:doi.org/10.1016/0006-291X(90)91362-V).
12. **Shiels, H. A., Vornanen, M. & Farrell, A. P.** 2002 Effects of temperature on intracellular $[Ca^{2+}]$ in trout atrial myocytes. *J Exp Biol* **205**, 3641-3650.
