# Supplementary material for: Developmental plasticity of cardiac anoxia-tolerance in juvenile common snapping turtles (Chelydra serpentina)
Source: Proc Biol Sci. 2019 Jun 26;286(1905):20191072. doi: 10.1098/rspb.2019.1072 (PMC6599983; doi:10.1098/rspb.2019.1072)
Supplement: Supplemental Results [file rspb20191072supp2.pdf]

2. Results

a) Effect of chronic developmental hypoxia on cardiomyocyte morphometrics

Cardiomyocytes from the H10 vs. N21 cohort were significantly smaller with a reduced length, depth, and volume, despite having larger widths (Tables 1 and S2).

(b) Effect of developmental O_2 and experimental treatment

The mixed-effects, repeated-measures GLMs revealed a significant effect of developmental O_2 on $\Delta[Ca^{2+}]_i$ and cell-shortening efficiency; an effect of treatment on cell shortening, $\Delta[Ca^{2+}]_i$, pH_i, ROS production, normalized $\Delta[Ca^{2+}]_i$, systolic $[Ca^{2+}]_i$, time to rise, and time to half-decay; an interaction between normoxia and developmental O_2 on the $\Delta[Ca^{2+}]_i$ and cell-shortening efficiency; and an interaction between anoxia and developmental O_2 on cell shortening, pH_i, ROS production, and cell-shortening efficiency (Table S3). Test statistics for significant within-group and between-group pairwise contrasts are provided in Supplementary Tables S4 and S5.

(c) Myofilament Ca^{2+}-sensitivity

The observation that H10 cardiomyocyte shortening was either equal to, or larger, than the N21 cells despite smaller Ca^{2+} transients, suggested the H10 cohort had greater myofilament Ca^{2+}-sensitivity. Indeed, cardiomyocyte shortening was greater in H10 cells at any given $\Delta[Ca^{2+}]_i$, when compared to N21 cells (Fig. 4A, B). Regression lines fitted to the data revealed significant, positive correlations between $\Delta[Ca^{2+}]_i$ and contractility during normoxia and reoxygenation in N21 cardiomyocytes (but not anoxia) and during normoxia, anoxia, and reoxygenation in H10 cardiomyocytes (Fig. 4A, B). A GLM analysis revealed significant effects of developmental O_2, period (normoxia-anoxia), and developmental O_2-period interactions (N21 normoxia-N21 anoxia, N21 anoxia-H10 normoxia, N21 anoxia-H10 anoxia, and N21 anoxia-H10 reoxygenation). When comparing the estimated marginal means of the GLM, cardiomyocyte shortening was equal between the H10 cohort (normoxia, anoxia, and reoxygenation) and N21 normoxia and reoxygenation, but not N21 anoxia (Fig. 4A, B). These analyses show that H10 cardiomyocytes in anoxia shorten equally to all normoxia- or reoxygenation-exposed cells. Lastly, a GLM revealed a significant effect of $[Ca^{2+}]_e$ and a significant developmental O_2-$[Ca^{2+}]_e$ interaction, while the post-hoc tests showed that H10 cells were more sensitive to Ca^{2+} (Fig. 4C). Regression lines were fitted to the data and revealed significant, positive correlations between $[Ca^{2+}]_e$ and length of N21 and H10 cardiomyocytes (Fig. 4C). Collectively, our data suggest that the myofilaments of H10 cardiomyocytes have a higher sensitivity to Ca^{2+} than N21 cells (Fig. 4A, B, and C). Test statistics for these analyses are provided in Supplementary Tables S6 and S7s.

Table S2. Between-group test statistics, comparing the effect of developmental normoxia (N21) and chronic hypoxia (H10) on ventricular cardiomyocyte morphometrics.

Variable	Comparison	Test statistic	df	P-value
Length	N21-H10	t = 2.3	66	0.024
Depth	N21-H10	U = 65		0.033
Width	N21-H10	U = 58		0.015
Volume	N21-H10	U = 266		0.031

Test values were calculated from a Student's t-test (length) or Mann-Whitney rank-sum tests (depth, width, and volume).

Table S3. GLM test statistics, comparing the effects of developmental O_2 and experimental treatment on cardiomyocyte physiology.

Factor	Variable	t-value	df	P-value
Developmental O_2	$\Delta[Ca^{2+}]_i$	2.9	18	0.009
	Cell-shortening efficiency	-3	19	0.007
Treatment	Cell shortening	9.6	107	< 0.001
	$\Delta[Ca^{2+}]_i$	10.9	97	< 0.001
	pH_i	22.8	125	< 0.001
	ROS production	8.4	83	< 0.001
	Normalized $\Delta[Ca^{2+}]_i$	14.8	103	< 0.001
	Systolic $[Ca^{2+}]_i$	4.2	105	< 0.001
	Time to rise	-6.1	31	< 0.001
	Time to half-decay	-2.7	29	0.011
Developmental O_2-normoxia interaction	$\Delta[Ca^{2+}]_i$	4.1	20	< 0.001
	Cell-shortening efficiency	-3	19	0.007
Developmental O_2-anoxia interaction	Cell shortening	-2.3	21	0.033
	pH_i	2.4	22	0.028
	ROS production	2.88	16	0.011
	Cell-shortening efficiency	-2.7	36	0.012

Test values were calculated from mixed-effects, repeated-measures GLMs.

Table S4. Within-group test statistics of the estimated means of the interactive effects between developmental oxygen and experimental time/period on cardiomyocyte physiology

Variable	Developmental cohort	Time comparison (min)	t-value	df	P-value
Shortening	N21	5 - 15	4.4	106	< 0.001
		5 - 20	3.7	106	0.003
		5 - 25	4.6	107	< 0.001
		5 - 30	5.0	107	< 0.001
		10 - 15	4.1	106	0.001
		10 - 20	3.4	106	0.007
		10 - 25	4.4	107	< 0.001
		10 - 30	4.8	107	< 0.001
		35 - 15	4.5	108	< 0.001
		35 - 20	3.8	108	0.002
		35 - 25	4.8	107	< 0.001
		35 - 30	5.2	107	< 0.001
		40 - 15	4.5	108	< 0.001
		40 - 20	3.8	108	0.002
		40 - 25	4.7	108	< 0.001
		40 - 30	5.1	107	< 0.001
	H10	5 - 15	5.3	106	< 0.001
		5 - 20	2.9	106	0.045
		10 - 15	5.2	106	< 0.001
		10 - 20	2.9	106	0.050
		25 - 15	3.2	106	0.042
		30 - 15	3.4	106	0.024
		35 - 15	3.6	107	0.006
		40 - 15	3.7	107	0.004
$\Delta[Ca^{2+}]_i$	N21	5 - 15	6.5	96	< 0.001
		5 - 20	6.8	96	< 0.001
		5 - 25	6.8	96	< 0.001
		5 - 30	6.1	96	< 0.001
		10 - 15	8.4	96	< 0.001
		10 - 20	8.8	96	< 0.001
		10 - 25	8.8	96	< 0.001
		10 - 30	8.0	96	< 0.001
		35 - 15	7.6	96	< 0.001
		35 - 20	8.0	97	< 0.001
		35 - 25	8.0	97	< 0.001
		35 - 30	7.4	96	< 0.001
		40 - 15	7.9	96	< 0.001
		40 - 20	8.2	97	< 0.001

		40 - 25	8.2	97	< 0.001
		40 - 30	7.6	96	< 0.001
	H10	35 - 15	3.2	95	0.021
		35 - 20	3.5	95	0.011
		35 - 25	3.1	95	0.028
		35 - 30	3.2	95	0.024
pH_i	N21	5 - 15	6.5	124	< 0.001
		5 - 20	6.8	124	< 0.001
		5 - 25	6.8	124	< 0.001
		5 - 30	6.1	124	< 0.001
		10 - 15	8.4	124	< 0.001
		10 - 20	8.8	124	< 0.001
		10 - 25	8.8	124	< 0.001
		10 - 30	8.0	124	< 0.001
		35 - 15	7.6	125	< 0.001
		35 - 20	8.0	125	< 0.001
		35 - 25	8.0	125	< 0.001
		35 - 30	7.4	125	< 0.001
		40 - 15	7.9	125	< 0.001
		40 - 20	8.2	125	< 0.001
		40 - 25	8.2	125	< 0.001
		40 - 30	7.6	125	< 0.001
	H10	5 - 15	7.4	124	< 0.001
		5 - 20	7.6	124	< 0.001
		5 - 25	7.6	125	< 0.001
		5 - 30	7.3	125	< 0.001
		10 - 15	8.1	124	< 0.001
		10 - 20	8.3	124	< 0.001
		10 - 25	8.3	125	< 0.001
		10 - 30	8.0	125	< 0.001
		35 - 15	9.5	125	< 0.001
		35 - 20	9.7	125	< 0.001
		35 - 25	9.8	125	< 0.001
		35 - 30	9.5	125	< 0.001
		40 - 15	8.8	125	< 0.001
		40 - 20	9.0	125	< 0.001
		40 - 25	9.1	125	< 0.001
		40 - 30	8.8	125	< 0.001
ROS	N21	5 - 20	3.1	82	0.035
		5 - 25	3.4	82	0.015
		5 - 30	3.3	82	0.017

		10 - 25	3.1	82	0.035
		10 - 30	3.0	82	0.038
	H10	5 - 15	5.3	82	< 0.001
		5 - 20	5.8	83	< 0.001
		5 - 25	6.1	83	< 0.001
		5 - 30	6.4	83	< 0.001
		5 - 35	3.5	83	0.015
		5 - 40	3.7	83	0.009
		10 - 15	4.7	82	< 0.001
		10 - 20	5.2	83	< 0.001
		10 - 25	5.5	83	< 0.001
		10 - 30	5.8	83	< 0.001
		35 - 30	2.9	82	0.041
Normalized $\Delta[Ca^{2+}]_i$	N21	5 - 15	4.5	98	< 0.001
		5 - 20	5.4	98	< 0.001
		5 - 25	4.4	100	< 0.001
		5 - 30	5.0	100	< 0.001
		10 - 15	5.7	98	< 0.001
		10 - 20	6.6	98	< 0.001
		10 - 25	5.5	100	< 0.001
		10 - 30	6.1	100	< 0.001
		35 - 15	5.1	99	< 0.001
		35 - 20	5.9	101	< 0.001
		35 - 25	5.0	102	< 0.001
		35 - 30	5.6	100	< 0.001
		40 - 15	4.8	99	< 0.001
		40 - 20	5.6	101	< 0.001
		40 - 25	4.8	102	< 0.001
		40 - 30	5.3	100	< 0.001
	H10	5 - 15	4.1	100	0.001
		5 - 20	4.6	100	< 0.001
		5 - 25	4.1	100	0.001
		5 - 30	4.5	100	< 0.001
		10 - 15	5.3	100	< 0.001
		10 - 20	5.8	100	< 0.001
		10 - 25	5.3	100	< 0.001
		10 - 30	5.7	100	< 0.001
		35 - 15	6.6	97	< 0.001
		35 - 20	7.0	97	< 0.001
		35 - 25	6.6	97	< 0.001
		35 - 30	6.9	97	< 0.001

		40 - 15	5.0	98	< 0.001
		40 - 20	5.5	98	< 0.001
		40 - 25	5.0	98	< 0.001
		40 - 30	5.3	98	< 0.001
Diastolic $[Ca^{2+}]_i$	N21	5 - 35	-3.8	124	0.007
		5 - 40	-3.8	124	0.006
	H10	5 - 35	-3.6	124	0.013
		5 - 40	-4.4	124	0.001
		10 - 35	-3.3	124	0.036
		10 - 40	-4.0	124	0.002
Systolic $[Ca^{2+}]_i$	N21	5 - 35	-3.9	104	0.002
		5 - 40	-3.3	104	0.014
		35 - 15	4.0	104	0.002
		35 - 20	3.9	104	0.002
		35 - 25	3.2	104	0.019
		35 - 30	2.9	104	0.045
		40 - 15	3.4	104	0.010
		40 - 20	3.3	104	0.014
	H10	5 - 35	-3.6	103	0.012
		5 - 40	-3.8	103	0.008
		10 - 35	-3.2	103	0.038
		10 - 40	-3.4	103	0.027
		35 - 15	3.3	104	0.014
		35 - 20	2.9	104	0.048
		40 - 15	3.5	104	0.010
		40 - 20	3.0	104	0.035

**Variable**	**Developmental cohort**	**Time comparison (period)**	**t-value**	**df**	**P-value**
Time to rise	N21	N - A	-3.8	30	0.001
		R - A	-3.5	31	0.002
	H10	N - A	-4.6	32	< 0.001
		R - A	-3.2	30	0.003
Time to half-decay	N21	N - A	-2.3	29	0.044
	H10	N - A	-2.3	30	0.039

Test values were calculated from mixed-effects, repeated-measures GLMs, followed by sequential Sidak post-hoc tests. Abbreviations: N21 and H10 indicate embryonic development in 21% and 10% O_2, respectively; df, degrees of freedom; and N, A, and R indicate experimental periods normoxia, anoxia, and reoxygenation, respectively.

Table S5. Between-group test statistics of the estimated means of the interactive effects between developmental oxygen, treatment, and time on cardiomyocyte physiology

Variable	Treatment	Time (min)	Comparison	t-value	df	P-value
Shortening	Anoxia	25	N21 - H10	-2.6	55	0.013
		30	N21 - H10	-3.1	59	0.003
$\Delta[Ca^{2+}]_i$	Normoxia	5	N21 - H10	3.5	30	0.001
		10	N21 - H10	4.3	30	< 0.001
		35	N21 - H10	3.1	37	0.004
		40	N21 - H10	3.2	39	0.002
pH_i	Normoxia	10	N21 - H10	2.0	40	0.049
	Anoxia	30	N21 - H10	2.1	45	0.042
ROS	Anoxia	15	N21 - H10	2.1	36	0.043
		20	N21 - H10	2.2	38	0.031
		25	N21 - H10	2.3	38	0.028
		30	N21 - H10	2.5	38	0.016
Efficiency	Normoxia	5	N21 - H10	-2.4	124	0.019
	Anoxia	25	N21 - H10	-2.1	126	0.034
		30	N21 - H10	-3.8	126	< 0.001

Test values were calculated from mixed-effects, repeated-measures GLMs, followed by sequential Sidak post-hoc tests. Abbreviations: N21 and H10 indicate embryonic development in 21% and 10% O_2, respectively; df, degrees of freedom

Table S6. Regression and GLM test statistics of the effects of developmental O_2 and experimental treatment on cardiomyocyte shortening, as a function of the calcium transient ($\Delta[Ca^{2+}]_i$).

Developmental cohort	Regression	Treatment	Test statistic	P-value	R^2
N21	Shortening vs. $\Delta[Ca^{2+}]_i$	N	F(1, 13) = 6.19	0.027	0.323
		R	[F(1, 6) = 6.82	0.040	0.532
H10	Shortening vs. $\Delta[Ca^{2+}]_i$	N	F(1, 16) = 7.42	0.015	0.317
		A	F(1, 29) = 5.67	0.024	0.164
		R	F(1, 13) = 4.59	0.05	0.261

GLM Factor	Comparison	df	P-value
Developmental O_2	N21-H10	1	< 0.001
Experimental period	N-A	1	0.022
Developmental O_2-period interactions	N21 N-N21 A	1	0.008
	N21 A-H10 N	1	< 0.001
	N21 A-H10 A	1	< 0.001
	N21 A-H10 R	1	0.001

Test values were calculated from regression analyses and mixed-effects GLMs. Abbreviations: N21 and H10 indicate embryonic development in 21% and 10% O_2, respectively; N, A, and R indicate experimental periods normoxia, anoxia, and reoxygenation, respectively.

Table S7. Regression and GLM test statistics of the effect of extracellular calcium ($[Ca^{2+}]_e$) on relaxed cardiomyocyte length.

GLM component	Test statistic	P-value
$[Ca^{2+}]_e$ factor	F(1, 34) = 80.93	< 0.001
Developmental O_2-$[Ca^{2+}]_e$ interaction	F(1, 34) = 15.98	< 0.001
N21-H10 post-hoc comparison	t = 3.19	< 0.001

Regression	Developmental cohort	Test statistic	P-value	R^2
Length vs. $[Ca^{2+}]_e$	N21	F(1, 22) = 30.75	< 0.001	0.583
	H10	F(1, 22) = 34.72	< 0.001	0.612

Test values were calculated from regression analyses and mixed-effects GLMs. Abbreviations: N21 and H10 indicate embryonic development in 21% and 10% O_2, respectively.
